# Braces versus casts for post-operational immobilization of ankle fractures: A meta-analysis

**DOI:** 10.3389/fsurg.2022.1055008

**Published:** 2023-01-25

**Authors:** Bin Li, Jianying Xie, Zhengmao Zhang, Quanyong Liu, Jialie Xu, Chenxi Yang

**Affiliations:** Department of Orthopaedics, Yuhuan People’s Hospital, Taizhou, China

**Keywords:** cast, brace, ankle fractures, post operational, immobilization

## Abstract

**Background and aims:**

Both casts and braces can be used for post-operational immobilization of ankle fractures. This meta-analysis aimed to assess the complications and functional effects of the two types of immobilization.

**Material and methods:**

PubMed, Embase, Cochrane, and CNKI was searched for randomized controlled trials (published between Jan 1, 1950, and March 2022). Relative risk (RR) or standard mean difference (SMD) with a 95% confidence interval (CI) was used to present the outcomes. The pooled data were assessed by using the fixed-effects model or random-effects model.

**Results:**

A total of 5 randomized controlled studies involving 930 subjects were included according to our inclusion criteria. On the ankle score at 6w,12w and 52w, there was no statistically significant difference between the two groups. In terms of 6w, the brace group showed better ankle dorsiflexion (MD = 6.78, 95% CI 0.56–13.00, *p* = 0.03) and plantar flexion (MD = 6.58, 95% CI 1.60–11.55, *p* = 0.01) than the cast group. The wound complications (RR = 3.49, 95% CI 1.32 to 9.24, *p* = 0.01) and total complications (RR = 3.54, 95% CI 1.92 to 6.50, *p* < 0.0001) in the brace group were three times more than that in the cast group. There was no statistically significant difference between the two groups in the non-wound complications. There was no statistically significant difference between the two groups in the time of going back to work, swelling of the ankle, and atrophy of the calf muscle.

**Conclusion:**

The short-term and long-term functional outcomes after postoperative treatment of adult ankle fractures with braces are similar to those with casts. The usage of braces may cause three times more wound complications than that of casts.

## Introduction

As one of the most commonly seen fractures among humans, ankle fractures have an incidence ranging from 42.2 to 179/per 100,000 person-years (1–5), especially high in adolescent men and middle-aged and old women (1, 3, 4). The high incidence of ankle fractures may be associated with an aging population, increasing obesity rates, and widespread participation in physical activities (6). It greatly impacts society and individuals, increasing the medical and economic burden on society and individuals (7–9). Moreover, limb pain and reduced mobility caused by ankle fractures widely affect patients’ daily social activities, family life, and mental health (10).

Generally, cast immobilization is required for several weeks after an ankle fracture because the cast can provide maximum support for the fracture, promoting the healing of the fracture and decreasing the possibility of delayed union or nonunion of the fracture. However, prolonged ankle immobilization can bring a series of adverse effects, including muscle atrophy, stiffness of joint, joint pain, thrombosis in deep vein, and even some long-term impacts, such as abnormal gait and calf muscle weakness (11, 12). Detachable ankle joint braces, which have emerged in recent years, can avoid adverse consequences caused by the long-term immobilization of ankle joints and promote the early rehabilitation of patients. These braces can be worn freely, allowing patients to perform early rehabilitation activities, however, they may cause some adverse effects, such as wound infection, and dehiscence (11, 13, 14). Both methods are currently widely used in clinical practice. Nevertheless, which one is better for patients with ankle fractures remains debatable and inconclusive.

Therefore, this article aims to analyze the functional prognosis and complications of plaster casts and removable braces.

## Material and methods

Our research was reported strictly according to the Preferred Reporting Items for Systematic reviews and Meta-Analyses guidelines (15).

### Literature research and study selection

Pubmed, Cochrane, Embase, and CNKI electronic databases were retrieved to identify eligible publications describing the role of braces and casts in the post-operational immobilization published before March 2022. For Pubmed, the following The retrieval strategy used for Pubmed was shown in Appendix.

### Inclusion and exclusion criteria

Inclusion criteria: Comparative studies and RCTs comparing the clinical effects of casts versus braces for the treatment of adult ankle fractures (age ≥ 14 years) were eligible, and the outcomes included ankle scores (including Olerud and Molander ankle score, Mazur ankle score), complications (including wound complications and other complications), time of returning to work, motion range, swelling in the ankle, atrophy of the calf muscle. At the same time, eligible studies should provide sufficient data, including standard deviation and mean, for further extraction and pooling, and the number of participants grouped by different treatments for dichotomous and continuous outcomes. Exclusion criteria: retrospective studies; meeting abstracts; case reports; participants which are not adult ankle fractures; studies which cannot get available data.

### Study selection

Two researchers (JXi, ZZ) screened the abstracts and titles of all the selected studies in an independent way. The full text was downloaded if they failed to make a decision based on the abstract of a study. If there was any dissent, final decision was made through discussion and consensus with another reviewer (BL). The reasons why certain studies were excluded or ineligible were demonstrated.

### Quality assessment

The quality of the included studies were assessed by 2 researchers (QL, JXu) independently, during which the risk of bias assessment tool provided by the Cochrane collaboration in RevMan software was used. The quality assessment involved the following items: (1) selection bias: the generation of sequence and allocation concealment; (2) performance bias: blinding; (3) detection bias: incomplete data on the outcomes, (4) reporting bias: selective reporting of outcomes; and (5) other issues. There are three types of judgment: “Yes” (low risk of bias), “No” (high risk of bias), and “Unclear” (unclear or unknown risk of bias). Any dissent was resolved by consultation from a third reviewer (BL) for a final consensus.

### Data extraction

According to the Cochrane guidelines, a data collection spreadsheet was developed before data extraction, including publication information, type of study, patient number, age, follow-up time, outcome measures, and complications. Relevant data were extracted from all included studies by two researchers (BL, CY).

### Statistical analysis

The Cochrane Review Manager software, version 5.4.1, was employed in data analysis. The 95% confidence intervals and risk ratios were reported in the present research. *p* ≤ 0.05 demonstrated that there was a statistically significant difference. The pooled data was assessed by using the fixed-effects or random-effects model. The chi-square test was applied to explore the heterogeneity, with the statistical significance of *p* < 0.100. The index of *I*^2^ was used to quantify the heterogeneity, *I*^2^ <50% indicates low heterogeneity and we should choose fixed-effects model. *I*^2^ >50% indicates low heterogeneity and we should choose random-effects model.

## Results

### Descriptions of the included studies

**Figure 1:**
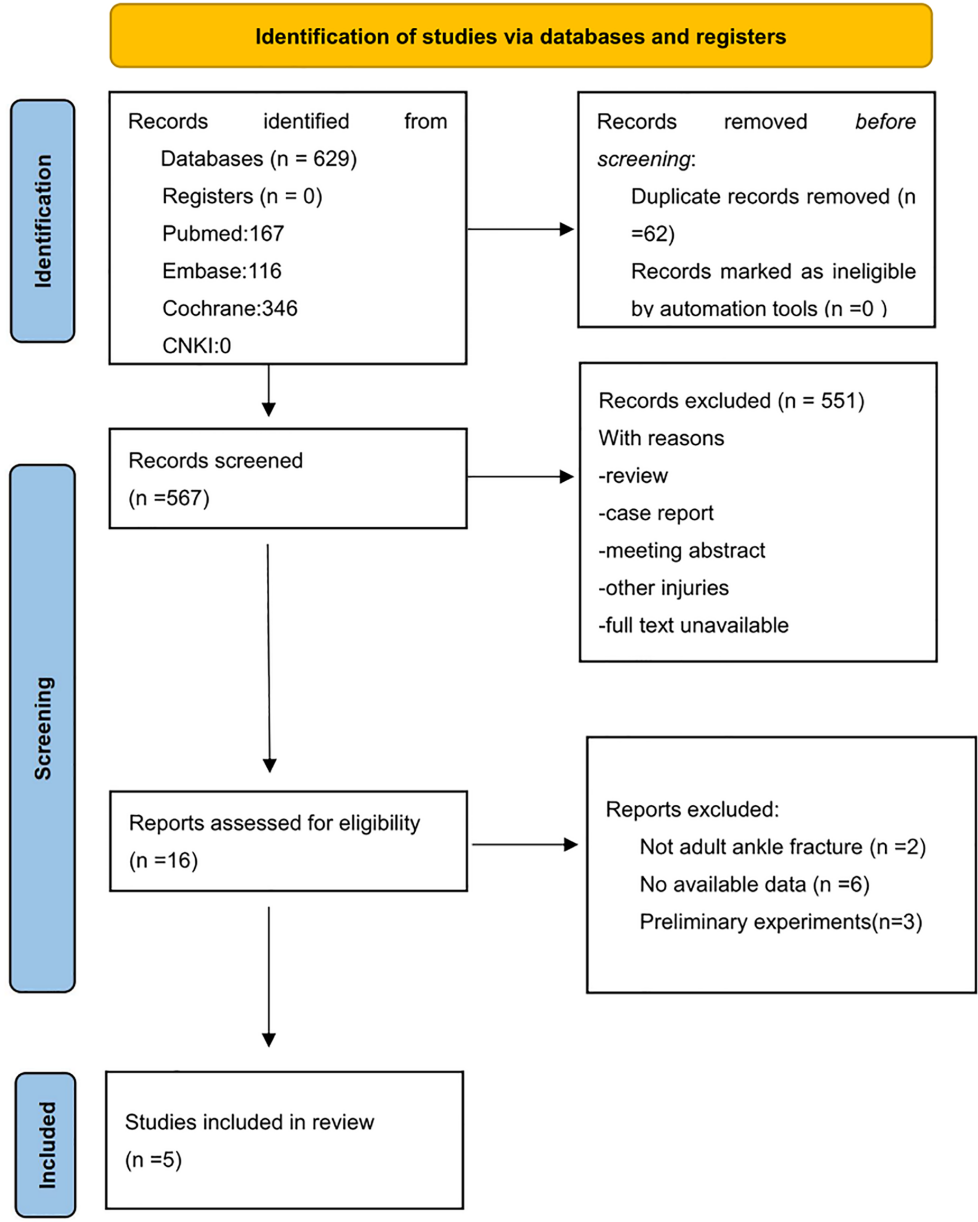
Flow diagram.

The PRISMA Flow Diagram was used to present the process of study selection (Figure 1). A total of 484 studies were searched from the aforementioned electronic databases, and 5 randomized control trials were eligible. All of the five eligible studies (13, 16–19) compared the two immobilization methods (casts and braces) for ankle fractures. These studies involved 930 patients, with 462 and 468 cases in the cast and brace group, respectively. Among the 5 eligible studies, 2 studies (18, 19) assessed the ankle score at 6w; three (17–19) rated the ankle score at 12w; two (18, 19) evaluated the ankle score at 52w; and two studies (17, 18) examined the time of returning to work, swelling of the ankle, and atrophy of the calf muscle. All of them provided data on complication rate and further details were shown in Table 1.

**Table 1:** Demographic features of the included articles.

Study	Study design	Country	Number (C/B)	Age (years) (C/B)	Intervention	Outcome	Follow up
Kearney 2021 (13)	RCT	UK	334/335	46.7 ± 17/45.9 ± 16	C/B	1,2,3,4,5	16W
Berg 2018 (16)	RCT	Netherlands	21/23	41.9 ± 16.3/44.3 ± 14.9	C/B	1,5,8,11	1Y
Vioreanu 2007 (17)	RCT	Ireland	29/33	34.9 ± 16/37.2 ± 12.9	C/B	1,5,6,7,8,9	6M
Lehtonen 2003 (18)	RCT	Finland	50/50	41 ± 13/41 ± 13	C/B	1,5,6,7,8,9	2Y
Egol 1999 (19)	RCT	USA	28/27	45.6 ± 17.5/39.5 ± 17.2	C/B	1,5,9,10	52w

C, cast group; B, brace group; 1, ankle score; 2, disability rating index (DRI); 3, self-administered health related quality of life measures (EQ-5D-5l); 4, Manchester-Oxford foot questionnaire (MOXFQ); 5, complications; 6, swelling of the ankle (mm); 7, atrophy of the calf muscle (mm); 8, range of motion; 9, return to work; 10, SF-36scores; 11, VAS score.

### Bias assessment

**Figure 2:**
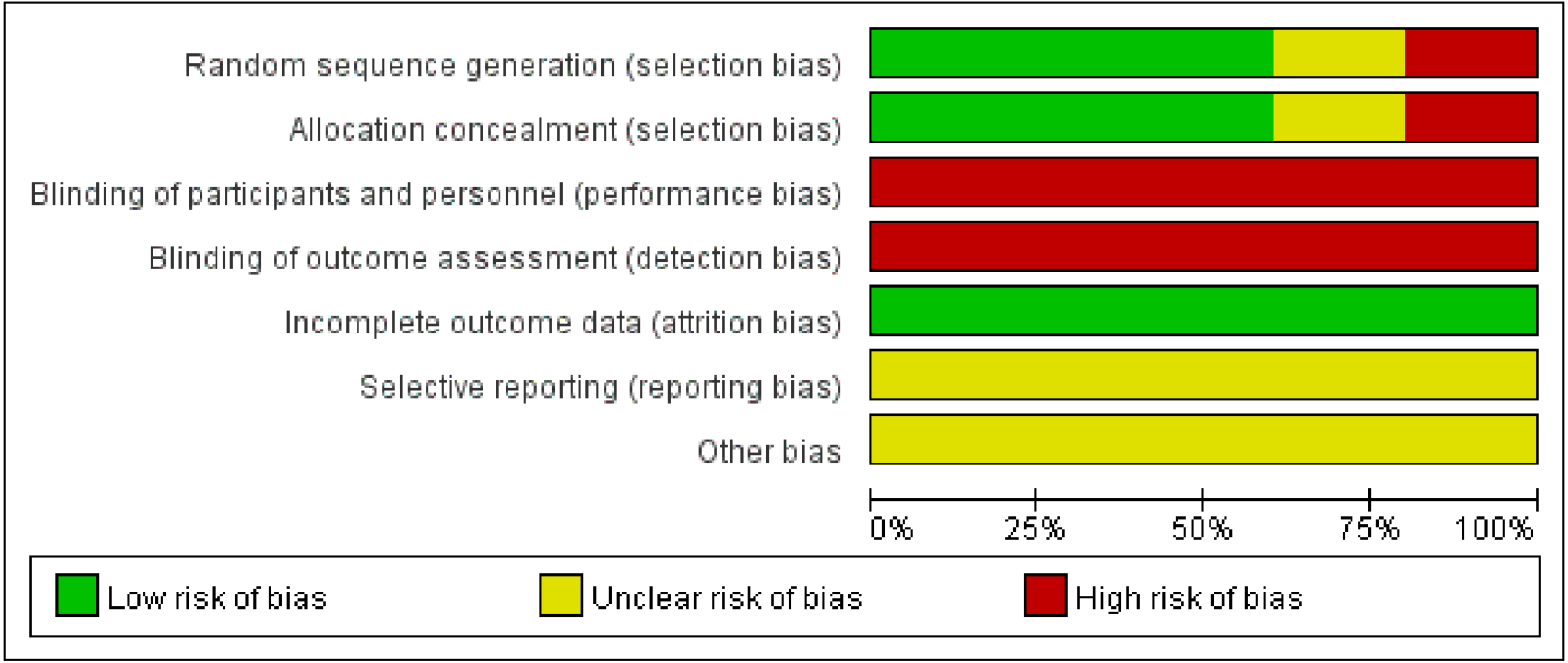
Map for the risk of bias: review the author's decision on the risk of bias items, expressed as a percentage of all the included articles.

**Figure 3:**
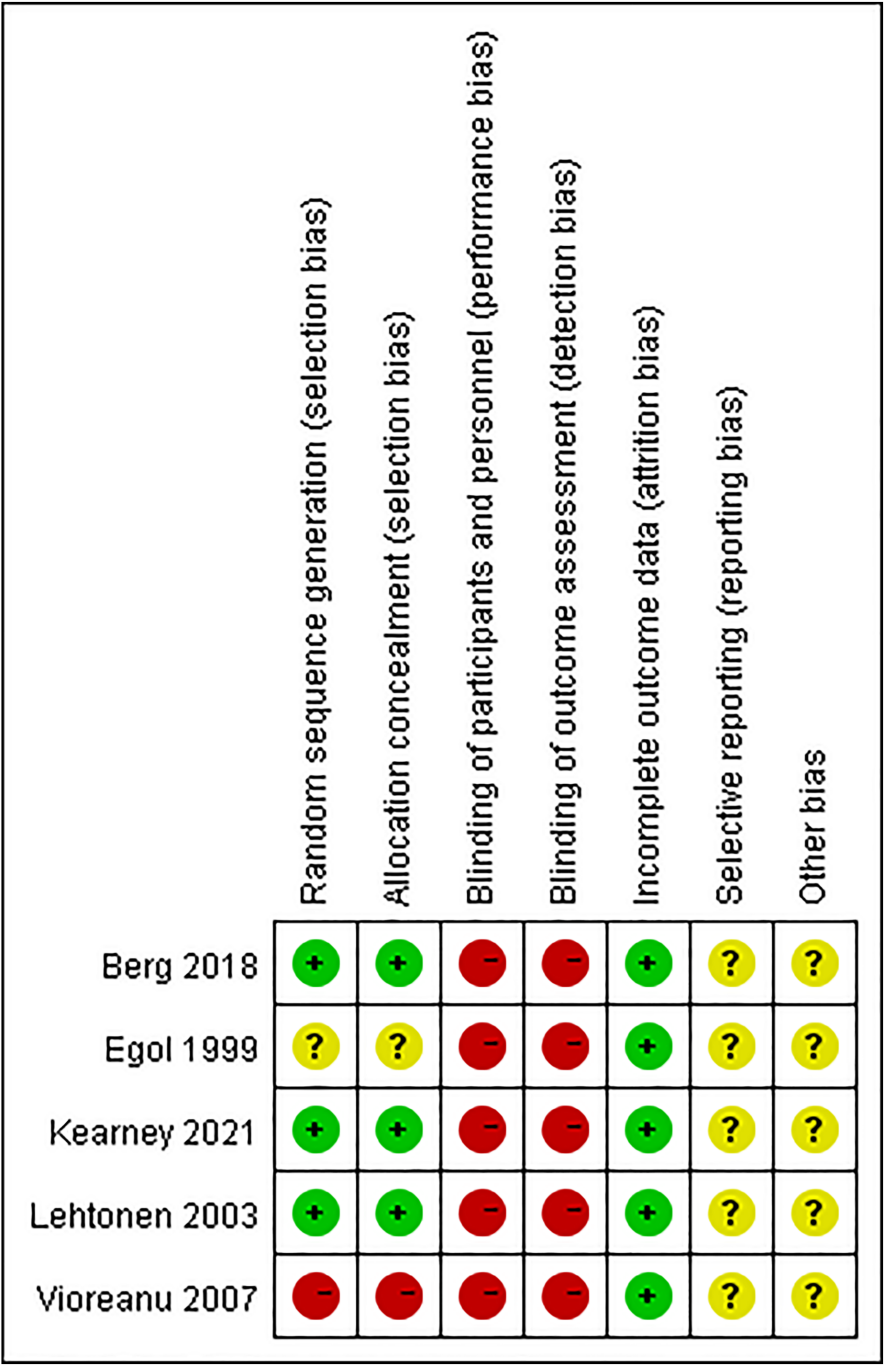
Summary of the risk of bias: review authors’ decision on the risk of bias item one by one for every included article.

Assessment of bias was performed for each of the five eligible RCTs, three of which (13, 16, 18) adopted adequate approach to obtain random sequence. For another two studies, one of them (17) used even or odd day of the date of birth, which revealed that selection bias was at high risk while the other one (19) only used “randomly,” which revealed that the risk of selection bias was unclear. Because the interventions in the five included studies were immobilization methods, it is challenging to apply blinding for research subjects and the staffs. The blinding of study participants and staffs was at high risk in the 5 studies, and the risk of selective reporting and other biases was unclear (Figures 2, 3).

### Ankle score

**Figure 4:**
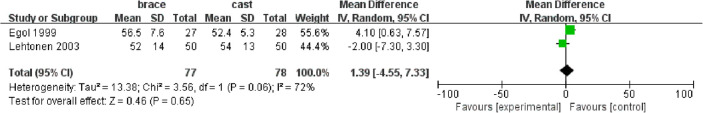
Forest plot of comparing cast versus brace groups for the 6w ankle score. CI, confidence interval.

Olerud Molander ankle score and Mazur score were used to assessing the ankle function. Two studies assessed the ankle scores at 6 and 52w; three studies assessed the ankle score at 12w. The random-effects model for the meta-analysis on the ankle score at 6w and 12w, and fixed-effects model for the meta-analysis on the ankle scores at 52w. The meta-analyses presented that there was no statistically significant difference on the ankle score at 6w (mean difference 1.39, 95% CI −4.55 to 7.33, *I*² = 72%, *p* = 0.06; figure 4), 12w (mean difference 5.56, 95% CI −1.71 to 12.84, *I*²= 83%, *p* = 0.13; Supplementary Figure S1) and 52w (mean difference 0.45, 95% CI −2.24 to 3.15, *I*² = 47%, *p* = 0.74; Supplementary Figure S2) between the brace and cast groups.

### Complications

**Figure 5:**
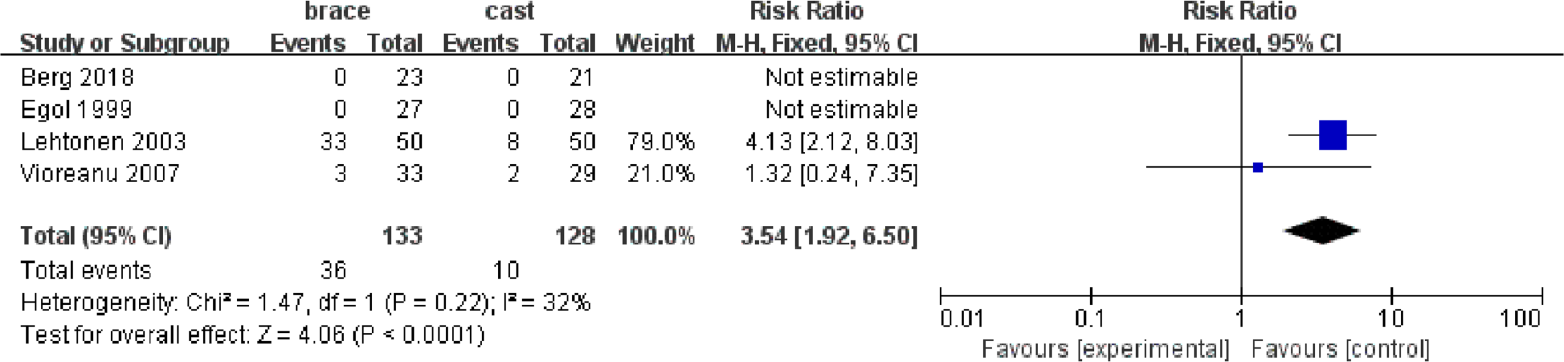
Forest plot of the comparison of cast versus brace groups for the wound complication. CI, confidence interval; M-H, Mantel-Haenszel test.

All included studies mentioned complications, two reporting no complications and the other three reporting complications, including wound complications and non-wound complications (such as deep vein thrombosis, chronic dysesthesias, chronic allodynia, and loss of internal fixation). However one study only reported wound complications (13). There was no markedly significant difference in non-wound complications (risk ratio 0.84, 95% CI 0.10 to 7.10, *I*² = 51%, *p* = 0.15; Supplementary Figure S4) between the two groups. However, the incidence of total complications (risk ratio 3.54, 95% CI 1.92 to 6.50, *I*² = 32%, *p* < 0.0001; Supplementary Figure S3) and wound complications (risk ratio 3.49, 95% CI 1.32 to 9.24, *I*² = 57%, *p* = 0.01; figure 5) in the brace group were higher compared with the cast group.

### Other outcomes

**Figure 6:**
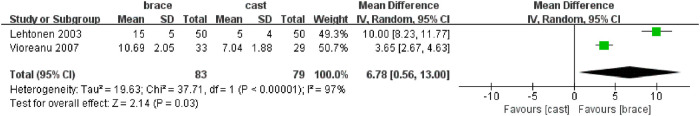
Forest plot of the comparison of cast versus brace groups for the ankle dorsiflexion in 6w. CI, confidence interval.

**Figure 7:**
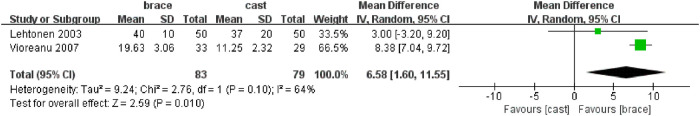
Forest plot of comparing cast versus brace groups for the ankle plantar flexion in 6w. CI, confidence interval.

Two studies reported time of returning to work, swelling and motion range of the ankle, and atrophy of the calf muscle. The random-effects model was only used in the meta-analysis on swelling of the ankle. Between the cast and brace groups, there was no statistically significant difference in the time of returning to work (mean difference −12.61, 95% CI −41.90 to 16.69, *I*^2^ = 95%, *p* = 0.40; Supplementary Figure S5), swelling of the ankle (mean difference −0.28, 95% CI −3.33 to 2.77, *I*^2^ = 0%, *p* = 0.86; Supplementary Figure S6), atrophy of the calf muscle (mean difference −9.59, 95% CI −39.81 to 20.63, *I*^2^ = 98%, *p* = 0.53; Supplementary Figure S7). However, the brace group showed better ankle dorsiflexion (mean difference 6.78, 95% CI 0.56–13.00, *I*^2^ = 97%, *p* = 0.03; Figure 6) and plantar flexion (mean difference 6.58, 95% CI 1.60–11.55, *I*^2^ = 64%, *p* = 0.01; Figure 7) than the cast group in 6w.

## Discussion

This meta-analysis revealed that the short-term and long-term functional outcomes after postoperative treatment of ankle fractures with braces are similar to those with casts. Although the ankle range of motion in the brace group is better compared with the cast group at the short term (6w). However, the total complications and wound complications in the brace group are three times more than that in the cast group. The non-wound complications were similar between the brace and cast groups. The time of returning to work, swelling of the ankle, and atrophy of the calf muscle show no remarkable difference between brace and cast groups.

Casts and braces have an obvious difference in biomechanical characteristics. One study (20) compared the capability of braces and casts in immobilizing the hindfoot and ankle. As tested, the results showed that casts showed better immobilizing effects in all directions compared with braces. However, casts are irremovable, which may constrain the ankle joint's early mobilization and hinder patients from observing and cleaning their limbs. Conversely, braces are removable and have properly fixed strength. Whether the braces would bring better clinical outcomes remains controversial.

Of the five eligible clinical trials comparing the two immobilization types, three trials (13, 16, 18) concluded that the function after treating ankle fracture using a cast is similar to using a functional brace. Some trails (17, 19) showed that the function was better at short-term follow-up. Our meta-analysis shows that the outcomes of ankle fracture adults using braces are similar to those with casts. Our results are different to previous reviews (14, 21).

Early ankle mobilization has been shown to restore the motion range of the joints, attenuate muscle atrophy (12), and prevent the progression of osteoporosis (22). This provides a theoretical basis for using a functional brace. A systematic review (21) concludes that motion at an early phase contributed to early return to work and improved motion range at 12w in comparison with using cast for immobilization. As mentioned, our study showed that the range of motion in the brace group was markedly improved compared with the cast group. However, this advantage can't transfer to more minor swelling of the ankle, milder atrophy of the calf muscle, or shorter time of returning to work. This difference may be attribute to few studies and higher heterogeneity in our meta-analysis.

Treating ankle fracture patients with a brace may improve the outcomes for a short time, but the complications should not be ignored. Two reviews (14, 21) conclude that using a brace has an elevated risk of infection in wounds compared with a cast. Our meta-analysis shows the total complications and wound complications in patients using braces are three times higher than those using casts. This outcome may be due to the early mobilization affecting the wounds or few soft tissues enveloping the ankle. This meta-analysis was the first to compare the brace and the cast for adult ankle fractures. There are still several limitations to this study. First, few trials are included in the present study. Of only five eligible studies, three studies have relatively small sample sizes. Second, the braces in these trials are different, which may influence the outcomes. Moreover, the technology of orthopedic internal fixation differs from each other, which may influence the stability of the ankle fracture after operation. One study (20) reported that the braces’ fixed strength depends on their conformity to foot shape and the properties of the material. Third, the follow-up time varies in each trial, from 16 weeks to 2 years. It might be possible that the longer the follow-up is, the more adverse events would occur, especially non-wound complications. At last, heterogeneity is statistically significant in the meta-analysis of some outcomes, such as time of returning to work as well as swelling and motion range of ankle. On the other hand, these studies didn't mention the costs or the comfort of the two types, which may influence patients’ final choice.

In conclusion, the functional outcomes after postoperative treatment of adult ankle fractures with braces may be similar to those with casts, no matter in the short-term or long-term. However, using a brace may cause three times wound complications more than a cast. Additional large randomized controlled trials are desired to validate our conclusions.

## Data Availability

The original contributions presented in the study are included in the article/**Supplementary Material**, further inquiries can be directed to the corresponding author.
