# Supplementary material for: Braces versus casts for post-operational immobilization of ankle fractures: A meta-analysis
Source: Front Surg. 2023 Jan 25;9:1055008. doi: 10.3389/fsurg.2022.1055008 (PMC9905617; doi:10.3389/fsurg.2022.1055008)
Supplement: Supplementary file 1 [file Datasheet1.pdf]

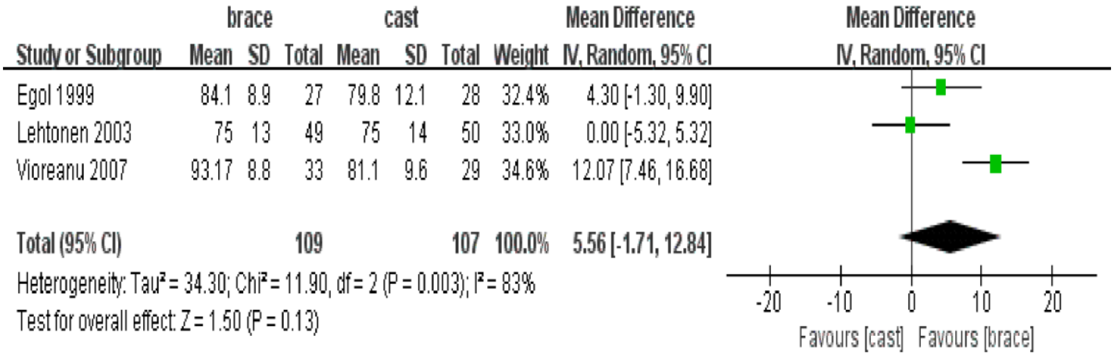

Figure S1. Forest plot of comparing cast versus brace groups for the 12w ankle score. CI, confidence interval

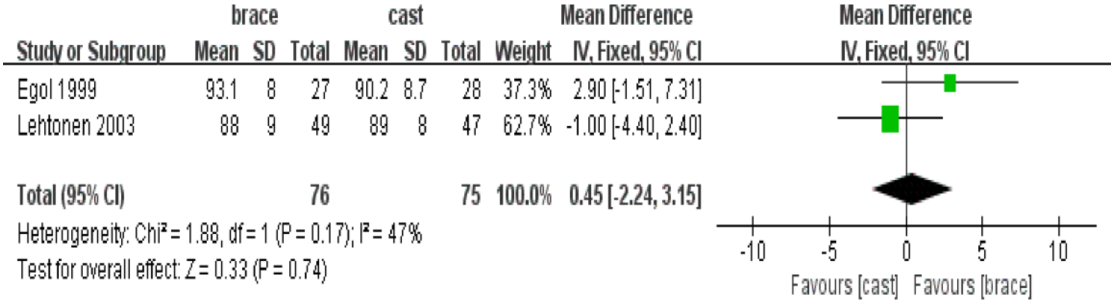

Figure S2. Forest plot of comparing cast versus brace groups for the 52w ankle score. CI, confidence interval

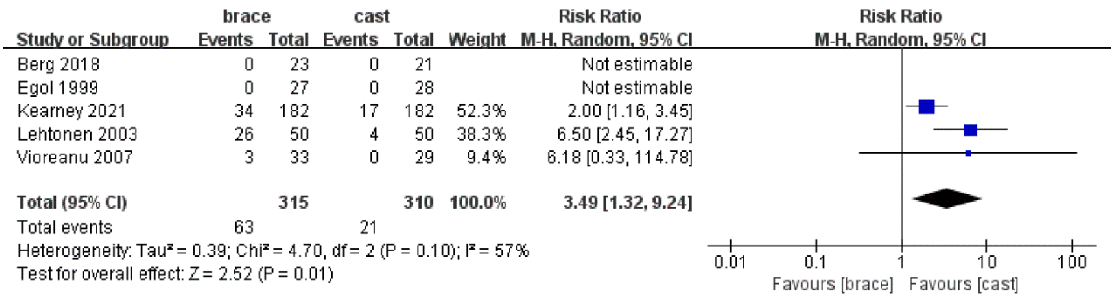

Figure S3. Forest plot of comparing cast versus brace groups for the total complication. CI, confidence interval; M-H, Mantel-Haenszel test

Study or Subgroup	brace Events	brace Total	cast Events	cast Total	Weight	Risk Ratio M-H, Random, 95% CI
Berg 2018	0	23	0	21		Not estimable
Egol 1999	0	27	0	28		Not estimable
Lehtonen 2003	7	50	4	50	68.2%	1.75 [0.55, 5.61]
Vioreanu 2007	0	33	2	29	31.8%	0.18 [0.01, 3.53]
**Total (95% CI)**		**133**		**128**	**100.0%**	**0.84 [0.10, 7.10]**
Total events	7		6			

Heterogeneity: Tau² = 1.38; Chi² = 2.02, df = 1 (P = 0.15); I² = 51%
Test for overall effect: Z = 0.16 (P = 0.88)

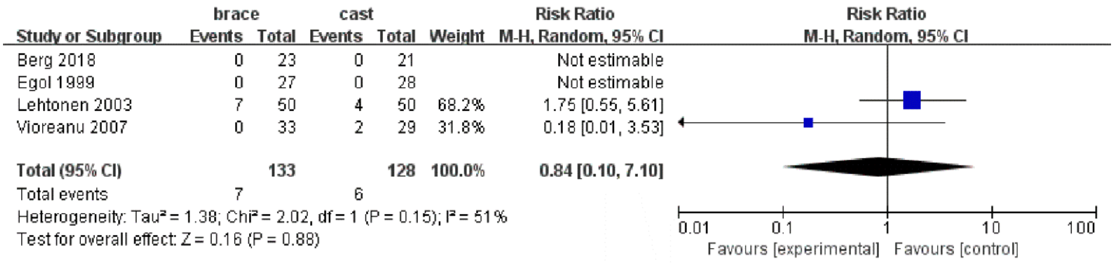

Figure S4. Forest plot of comparing cast versus brace groups for the other complication. CI, confidence interval; M-H, Mantel-Haenszel test

Study or Subgroup	brace Mean	brace SD	brace Total	cast Mean	cast SD	cast Total	Weight	Mean Difference IV, Random, 95% CI
Lehtonen 2003	65	19	50	63	13	50	51.1%	2.00 [-4.38, 8.38]
Vioreanu 2007	67	18.7	33	94.9	24.4	29	48.9%	-27.90 [-38.83, -16.97]
**Total (95% CI)**			**83**			**79**	**100.0%**	**-12.61 [-41.90, 16.69]**

Heterogeneity: Tau² = 426.14; Chi² = 21.43, df = 1 (P < 0.00001); I² = 95%
Test for overall effect: Z = 0.84 (P = 0.40)

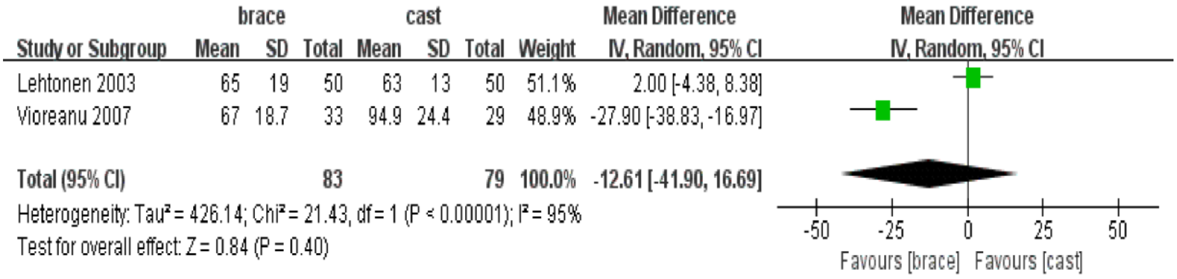

Figure S5. Forest plot of comparing cast versus brace groups for the time of returning to work. CI, confidence interval

Study or Subgroup	brace Mean	brace SD	brace Total	cast Mean	cast SD	cast Total	Weight	Mean Difference IV, Fixed, 95% CI
Lehtonen 2003	18	14	50	20	19	50	21.8%	-2.00 [-8.54, 4.54]
Vioreanu 2007	18.7	6.2	33	18.5	7.5	29	78.2%	0.20 [-3.25, 3.65]
**Total (95% CI)**			**83**			**79**	**100.0%**	**-0.28 [-3.33, 2.77]**

Heterogeneity: Chi² = 0.34, df = 1 (P = 0.56); I² = 0%
Test for overall effect: Z = 0.18 (P = 0.86)

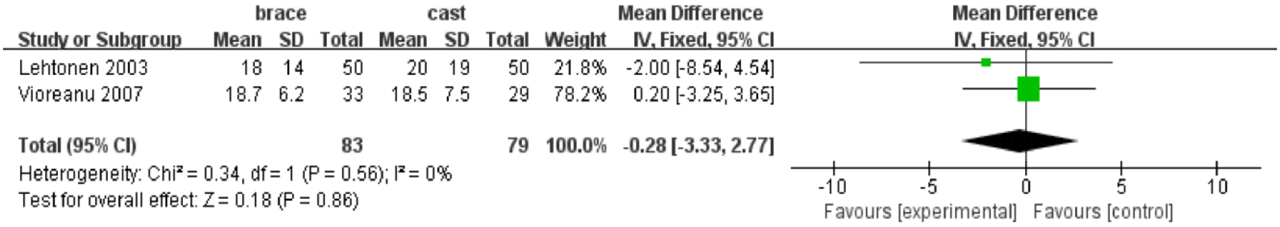

Figure S6. Forest plot of comparing cast versus brace groups for the swelling of the ankle. CI, confidence interval

Study or Subgroup	brace Mean	brace SD	brace Total	cast Mean	cast SD	cast Total	Weight	Mean Difference IV, Random, 95% CI
Lehtonen 2003	-4	12	50	-10	24	50	49.4%	6.00 [-1.44, 13.44]
Vioreanu 2007	10.16	9.64	33	35	5.4	29	50.6%	-24.84 [-28.67, -21.01]
**Total (95% CI)**			**83**			**79**	**100.0%**	**-9.59 [-39.81, 20.63]**

Heterogeneity: Tau² = 466.44; Chi² = 52.20, df = 1 (P < 0.00001); I² = 98%
Test for overall effect: Z = 0.62 (P = 0.53)

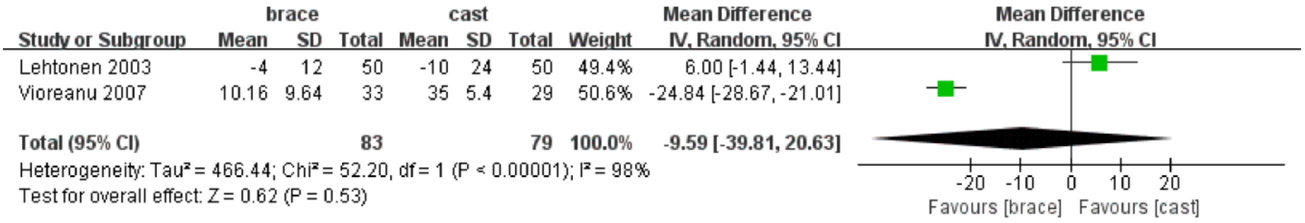

Figure S7. Forest plot of comparing cast versus brace groups for the atrophy of the

calf muscle. CI, confidence interval
