# Supplementary material for: Braces versus casts for post-operational immobilization of ankle fractures: A meta-analysis
Source: Front Surg. 2023 Jan 25;9:1055008. doi: 10.3389/fsurg.2022.1055008 (PMC9905617; doi:10.3389/fsurg.2022.1055008)
Supplement: Supplementary file 2 [file Datasheet2.docx]

**Appendix:**

search strategy for pubmed: (((((((((((((((((Surgical Casts) OR (Cast, Surgical)) OR (Surgical Cast)) OR (Plastic Casts)) OR (Cast, Plastic)) OR (Casts, Plastic)) OR (Plastic Cast)) OR (Plaster Casts)) OR (Cast, Plaster)) OR (Casts, Plaster)) OR (Plaster Cast)) OR (Fiberglass Casts)) OR (Cast, Fiberglass)) OR (Casts, Fiberglass)) OR (Fiberglass Cast)) OR ("Casts, Surgical"[Mesh])) AND ((((((((((((((((((((((((((((((((((((((((((((((((Ankle Fracture) OR (Fracture, Ankle)) OR (Fractures, Ankle)) OR (Lateral Malleolus Fractures)) OR (Fracture, Lateral Malleolus)) OR (Fractures, Lateral Malleolus)) OR (Lateral Malleolus Fracture)) OR (Malleolus Fracture, Lateral)) OR (Malleolus Fractures, Lateral)) OR (Trimalleolar Fractures)) OR (Fracture, Trimalleolar)) OR (Fractures, Trimalleolar)) OR (Trimalleolar Fracture)) OR (Trimalleolar Ankle Fractures)) OR (Ankle Fracture, Trimalleolar)) OR (Ankle Fractures, Trimalleolar)) OR (Fracture, Trimalleolar Ankle)) OR (Fractures, Trimalleolar Ankle)) OR (Trimalleolar Ankle Fracture)) OR (Medial Malleolus Fractures)) OR (Fracture, Medial Malleolus)) OR (Fractures, Medial Malleolus)) OR (Malleolus Fracture, Medial)) OR (Malleolus Fractures, Medial)) OR (Medial Malleolus Fracture)) OR (Posterior Malleolus Fractures)) OR (Fracture, Posterior Malleolus)) OR (Fractures, Posterior Malleolus)) OR (Malleolus Fracture, Posterior)) OR (Malleolus Fractures, Posterior)) OR (Posterior Malleolus Fracture)) OR (Bimalleolar Fractures)) OR (Bimalleolar Fracture)) OR (Fracture, Bimalleolar)) OR (Fractures, Bimalleolar)) OR (Bimalleolar Equivalent Ankle Fractures)) OR (Bimalleolar Equivalent Fractures)) OR (Bimalleolar Equivalent Fracture)) OR (Equivalent Fracture, Bimalleolar)) OR (Equivalent Fractures, Bimalleolar)) OR (Fracture, Bimalleolar Equivalent)) OR (Fractures, Bimalleolar Equivalent)) OR (Bimalleolar Ankle Fractures)) OR (Ankle Fracture, Bimalleolar)) OR (Ankle Fractures, Bimalleolar)) OR (Bimalleolar Ankle Fracture)) OR (Fracture, Bimalleolar Ankle)) OR (Fractures, Bimalleolar Ankle))) OR ((((((brace) OR (bracing)) OR (ankle support))) OR ("Braces"[Mesh])) AND (((((((((((((((((((((((((((((((((((((((((((((((((Ankle Fracture) OR (Fracture, Ankle)) OR (Fractures, Ankle)) OR (Lateral Malleolus Fractures)) OR (Fracture, Lateral Malleolus)) OR (Fractures, Lateral Malleolus)) OR (Lateral Malleolus Fracture)) OR (Malleolus Fracture, Lateral)) OR (Malleolus Fractures, Lateral)) OR (Trimalleolar Fractures)) OR (Fracture, Trimalleolar)) OR (Fractures, Trimalleolar)) OR (Trimalleolar Fracture)) OR (Trimalleolar Ankle Fractures)) OR (Ankle Fracture, Trimalleolar)) OR (Ankle Fractures, Trimalleolar)) OR (Fracture, Trimalleolar Ankle)) OR (Fractures, Trimalleolar Ankle)) OR (Trimalleolar Ankle Fracture)) OR (Medial Malleolus Fractures)) OR (Fracture, Medial Malleolus)) OR (Fractures, Medial Malleolus)) OR (Malleolus Fracture, Medial)) OR (Malleolus Fractures, Medial)) OR (Medial Malleolus Fracture)) OR (Posterior Malleolus Fractures)) OR (Fracture, Posterior Malleolus)) OR (Fractures, Posterior Malleolus)) OR (Malleolus Fracture, Posterior)) OR (Malleolus Fractures, Posterior)) OR (Posterior Malleolus Fracture)) OR (Bimalleolar Fractures)) OR (Bimalleolar Fracture)) OR (Fracture, Bimalleolar)) OR (Fractures, Bimalleolar)) OR (Bimalleolar Equivalent Ankle Fractures)) OR (Bimalleolar Equivalent Fractures)) OR (Bimalleolar Equivalent Fracture)) OR (Equivalent Fracture, Bimalleolar)) OR (Equivalent Fractures, Bimalleolar)) OR (Fracture, Bimalleolar Equivalent)) OR (Fractures, Bimalleolar Equivalent)) OR (Bimalleolar Ankle Fractures)) OR (Ankle Fracture, Bimalleolar)) OR (Ankle Fractures, Bimalleolar)) OR (Bimalleolar Ankle Fracture)) OR (Fracture, Bimalleolar Ankle)) OR (Fractures, Bimalleolar Ankle)) OR ("Ankle Fractures"[Mesh]))) AND (clinicaltrial[Filter] OR randomizedcontrolledtrial[Filter]).
